# Changes in sleep quality amidst COVID-19 pandemic among psychiatric patients in Romania

**DOI:** 10.1192/j.eurpsy.2021.729

**Published:** 2021-08-13

**Authors:** M. Petrescu, A. Mitrea, C. Tudor, D. Vasile

**Affiliations:** Psychiatry, University Emergency Central Military Hospital Dr. Carol Davila, Bucharest, Romania

**Keywords:** COVID-19, sleep quality, Insomnia

## Abstract

**Introduction:**

Sleep disturbances can occur as a result of major stressful events. Additionally, research evidence suggests that COVID-19 pandemic may have negatively impacted the quality of sleep among various populations. However, individuals respond differently to the stress, uncertainty and social isolation related with COVID-19 pandemic.

**Objectives:**

This study aimed to explore the changes in sleep quality and pattern among voluntary psychiatric patients visiting our clinic in Romania during COVID-19 pandemic.

**Methods:**

We implemented a cross-sectional study over a period of 3 months, utilizing a Romanian-translated version of the Pittsburgh Sleep Quality Index (PSQI) which was administered through Google Forms web application. Participants lacking digital skills were provided with guidance for completing the questionnaire. Informed consent was obtained prior to participating in this study and data anonymity and confidentiality were ensured.

**Results:**

Among a total of 98 responders, 63% reported a global PSQI score greater than 5, indicating poor sleep. Approximately 25% of participants subjectively marked their sleep as either fairly bad or very bad. When analysing the 7 components of PSQI, our participants struggled most with long sleep latency. About a third of participants reported using sleep medication (both prescription and over-the-counter) three or more times a week within the past month.

**Conclusions:**

Considering the fact that the current situation is likely to evolve for an unknown period of time, there is a dire need to assess the effect of prolonged adjustments in daily routine and their impact on the sleep and the quality of life of our patients.

